# Robust 3D Multi-Object Tracking via 4D mmWave Radar-Camera Fusion and Disparity-Domain Depth Recovery

**DOI:** 10.3390/s26072096

**Published:** 2026-03-27

**Authors:** Yunfei Xie, Xiaohui Li, Dingheng Wang, Zhuo Wang, Shiliang Li, Jia Wang, Zhenping Sun

**Affiliations:** 1 Northwest Institute of Mechanical and Electrical Engineering, Xianyang 712099, China; xyf8951@nudt.edu.cn (Y.X.);; 2 College of Intelligence Science and Technology, National University of Defense Technology, Changsha 410073, China; xiaohui_lee@outlook.com

**Keywords:** 4D millimeter-wave radar, monocular depth estimation, disparity-domain recalibration, multi-object tracking, sensor fusion

## Abstract

4D millimeter-wave radar provides high-precision ranging capability and exhibits strong robustness under adverse weather and low-visibility conditions, but its point clouds are relatively sparse and suffer from severe elevation-angle measurement noise. Monocular cameras, by contrast, provide rich semantic information and high recall, yet are fundamentally limited by scale ambiguity. To exploit the complementary characteristics of these two sensors, this paper proposes a radar-camera fusion 3D multi-object tracking framework that does not rely on complex 3D annotated data. First, on the radar signal-processing side, a Gaussian distribution-based adaptive angle compression method and IMU-based velocity compensation are introduced to effectively suppress measurement noise, and an improved DBSCAN clustering scheme with recursive cluster splitting and historical static-box guidance is employed to generate high-quality radar detections. Second, a disparity-domain metric depth recovery method is proposed. This method uses filtered radar points as sparse metric anchors, performs robust fitting with RANSAC, and applies Kalman filtering for temporal smoothing, thereby converting the relative depth output of the visual foundation model Depth Anything V2 into metric depth. Finally, a hierarchical fusion strategy is designed at both the detection and tracking levels to achieve stable cross-modal state association. Experimental results on a self-collected dataset show that the proposed method achieves an overall MOTA of 77.93%, outperforming single-modality baselines and other comparison methods by 11 to 31 percentage points. This study provides an effective solution for low-cost and robust environment perception in complex dynamic scenarios.

## 1. Introduction

Environmental perception is a core function of autonomous driving systems, whose objective is to detect and continuously track surrounding traffic participants accurately and in real time in complex dynamic traffic scenarios. Current production-level perception systems commonly employ multi-sensor configurations that combine LiDAR and cameras. LiDAR provides high-precision 3D point clouds, but its high cost and pronounced performance degradation under adverse weather conditions such as rain, fog, and snow have limited its widespread adoption in mass-produced vehicles [1]. By contrast, monocular cameras are low-cost and easy to deploy, and they provide rich texture and semantic information. However, due to perspective projection, monocular imagery suffers from inherent scale ambiguity in depth, making it difficult to directly recover the true 3D positions of objects, especially at long range where scale drift and localization instability often occur [2].

The emergence of 4D imaging millimeter-wave radar has created new opportunities for building low-cost 3D perception systems with improved robustness under adverse weather conditions. Compared with conventional 3D radar, 4D radar incorporates vertical antenna arrays to measure elevation angles and can output four-dimensional point cloud data containing range, azimuth, elevation, and Doppler velocity [3]. Millimeter-wave radar exhibits stronger penetration capability in adverse weather conditions and is substantially less expensive than LiDAR, making it an attractive sensing modality for adverse-weather and low-visibility scenarios [4]. Nevertheless, because of limitations in antenna-array size, angular resolution, and multipath effects, 4D radar point clouds remain relatively sparse and exhibit substantial measurement noise in the vertical direction, often producing scattered points and false echoes [5]. Meanwhile, recent monocular depth estimation models have made significant progress in cross-scene generalization and can recover fine-grained relative depth structures from a single image [6,7,8,9]. Therefore, if the sparse metric information provided by 4D radar can be used to assign physical scale to monocular relative depth, one may combine the semantic expressiveness of vision with the ranging and velocity-measurement capability of radar, thereby improving 3D perception and tracking in complex scenarios.

Existing studies on monocular metric depth recovery and radar-vision fusion have explored several directions. For depth recovery, researchers have attempted to use sparse LiDAR or millimeter-wave radar observations to provide metric constraints for monocular depth, thereby converting relative depth into true depth [10,11,12,13]. For fusion detection, CenterFusion [14], RCFusion [15], and SGDet3D [16] have improved radar-camera 3D detection through frustum association, BEV feature fusion, and joint semantic-geometric modeling, respectively. For multi-object tracking, RaTrack [17], Tang et al. [18], and Kuan et al. [19] further explored the complementary strengths of 4D radar and vision for target association and trajectory maintenance, showing that cross-modal fusion is valuable for robust tracking in dynamic scenes.

Despite these advances, the emphases of existing approaches remain different. First, 4D radar-based detection and tracking methods can make full use of range, Doppler, and temporal point-cloud information, showing good potential in moving-object detection, state estimation, and online association [17,20,21]. However, owing to the sparsity of 4D radar point clouds, they still require visual information to complement fine-grained semantic understanding and geometric characterization. Second, existing radar-assisted monocular depth recovery methods have shown that sparse radar observations can provide effective metric constraints for monocular depth estimation [12,13]; however, under complex scenarios, how to further improve long-range scale consistency, suppress outlier interference, and enhance the stability of cross-modal correspondences remains an open problem. Third, many radar-camera fusion methods focus primarily on end-to-end 3D detection or feature-level fusion and usually depend on large-scale 3D annotated data [14,15,16,22]. In contrast, studies on using 4D radar static points to perform online metric recalibration of monocular relative depth, and then exploiting the resulting metric depth for subsequent 3D multi-object tracking, are still relatively scarce. Therefore, how to exploit the physical measurement properties of 4D radar to achieve robust monocular metric depth recovery without relying on complex 3D annotations, and how to translate this recovery into stable geometric observations for both detection-layer fusion and tracking-layer association, remains a worthwhile problem.

To address these issues, this paper proposes a 3D multi-object tracking framework based on 4D millimeter-wave radar and monocular camera fusion. Under known radar-camera extrinsic calibration, the framework uses 4D radar static points to provide sparse metric anchors for the relative depth predicted by Depth Anything V2, and achieves monocular metric depth recovery through disparity-domain robust fitting and temporal smoothing. Meanwhile, the radar branch generates stable radar observations through elevation-noise suppression, ego-motion compensation, and point-cloud clustering. Finally, cross-modal fusion and trajectory association are performed separately at the detection and tracking layers, enabling stable 3D multi-object tracking in complex dynamic scenarios.

The main contributions of this paper are as follows:

- An enhanced detection pipeline for 4D millimeter-wave radar point clouds is proposed. By combining Gaussian distribution-based elevation-noise suppression, IMU-based velocity compensation, and a recursive cluster splitting strategy with geometric constraints, the pipeline effectively alleviates multipath interference and over-clustering, thereby improving the geometric accuracy and temporal stability of radar detections.
- A monocular metric depth recovery method anchored by 4D radar static points is proposed. The method performs robust fitting in the disparity domain using RANSAC with adaptive MAD thresholds and applies Kalman filtering to temporally smooth the calibration parameters, thereby improving long-range scale consistency and robustness to outliers.
- A radar-camera fusion multi-object tracking framework with decoupled detection and tracking layers is constructed. The framework fuses visual semantics with radar motion information at the detection layer and uses a linear Kalman filter and the Hungarian algorithm for stable association at the tracking layer. Experiments on a self-collected dataset demonstrate that the proposed method outperforms single-modality baselines and traditional fusion baselines in both localization accuracy and tracking performance.

## 2. Related Work

### 2.1. Monocular Depth Estimation and Metric Recovery

Monocular depth estimation aims to recover 3D scene depth from a single RGB image. Because monocular images lack stereo disparity information, inferring 3D depth from 2D imagery is inherently ambiguous in scale, making accurate depth recovery a challenging task. In recent years, deep learning-based methods have achieved remarkable progress. Eigen et al. [2] first applied convolutional neural networks to monocular depth estimation, pioneering the data-driven depth prediction paradigm. MiDaS [6] was trained on mixed datasets and achieved excellent zero-shot cross-domain transfer capability, while also revealing that the output of monocular depth networks exhibits a linear relationship with true depth in the disparity domain. Ranftl et al. [7] proposed DPT, which introduced the Vision Transformer into dense prediction and significantly improved depth-estimation accuracy. Depth Anything [8] leveraged large-scale unlabeled data for self-supervised pretraining and combined it with knowledge distillation, achieving state-of-the-art performance on multiple benchmarks. Its improved version, Depth Anything V2 [9], further optimized the architecture and training strategy, improving boundary sharpness and detail preservation while maintaining efficient inference. However, these methods generally output relative or scale-free depth and cannot directly provide metric depth. To address scale recovery, researchers have explored multiple sensor-fusion strategies. In LiDAR-assisted depth estimation, Ma and Karaman [10] proposed a self-supervised sparse-to-dense depth completion framework that uses sparse LiDAR points to guide monocular depth learning. Park et al. [11] designed the non-local spatial propagation network, which achieves high-quality depth completion by learning affinity matrices. However, the high cost of LiDAR limits its large-scale deployment. Compared with LiDAR, millimeter-wave radar is lower cost and robust in all weather, and radar-assisted depth estimation has therefore attracted increasing attention. Long et al. [12] proposed the radar-camera pixel-level depth association method, which establishes correspondences between radar points and image pixels and fits depth transformation parameters using least squares. However, because this method performs linear fitting in the depth domain, and because the relationship between network outputs and true depth is nonlinear, it tends to incur large errors for distant objects and is sensitive to radar noise and outliers. Lin et al. [13] studied fusion strategies between sparse radar data and monocular images and proposed a confidence-weighted fusion scheme, but it did not fully exploit the theoretical advantages of the disparity domain and lacked a robust mechanism for suppressing outliers.

### 2.2. Radar Object Detection and Tracking

Millimeter-wave radar has become an important component of autonomous driving perception systems because of its all-weather capability, direct velocity measurement, and relatively low cost. Traditional automotive millimeter-wave radar can only provide range, azimuth, and radial velocity, and lacks the ability to measure height, which limits its use in complex scenarios. In recent years, the emergence of 4D millimeter-wave radar has brought new opportunities for radar perception. By adding vertical antenna arrays, 4D radar measures elevation and can output four-dimensional point cloud data containing range, azimuth, elevation, and velocity [3]. Compared with conventional 3D radar, 4D radar offers denser point clouds and improved angular resolution, making more refined object detection possible. However, 4D radar point clouds are still sparse compared with LiDAR and are strongly affected by multipath, especially in urban canyons and other complex environments where ground reflections and inter-vehicle reflections can produce many false targets. In radar point-cloud processing, clustering is the foundation of object detection. Palffy et al. [4] systematically evaluated multiple 4D radar point-cloud clustering methods on the View-of-Delft dataset and showed that density-based methods such as DBSCAN are suitable for radar point-cloud segmentation. Tan et al. [23] proposed a 3D object detection method for multi-frame 4D radar point clouds, improving detection performance through temporal information fusion. Shi et al. [24] proposed a moving least-squares-enhanced 3D object detection method that effectively handles point-cloud sparsity. Nevertheless, most existing methods cluster raw radar point clouds directly and lack preprocessing steps that explicitly address multipath effects and long-range sparsity, which leads to false targets and degraded clustering quality. In multi-object tracking, Pan et al. [17] proposed RaTrack, which combines spatial point-cloud features and Doppler information to detect and track moving objects. Tan et al. [20] proposed a 4D radar multi-object tracking method for urban environments that achieves more accurate trajectory estimation by distinguishing static and dynamic objects. Liu et al. [21] systematically compared several tracking frameworks for 4D imaging radar and reported that a Kalman filter combined with the Hungarian algorithm performs stably. However, existing methods mainly depend on radar measurements alone and therefore have difficulty estimating fine-grained object categories and geometric dimensions, which motivates the exploration of radar-camera fusion schemes for complementary perception.

### 2.3. Radar-Camera Fusion and Multi-Object Tracking

The fusion of cameras and millimeter-wave radar aims to combine the semantic perception capability of vision with the ranging and velocity-measurement advantages of radar, thereby enabling high-precision object detection and tracking. Radar-camera fusion methods typically rely heavily on accurate intrinsic and extrinsic calibration to accomplish cross-modal geometric alignment and projection. To address this issue, Cheng et al. [25] proposed a target-based calibration method using corner reflectors, achieving high-precision extrinsic estimation by solving a PnP problem combined with RANSAC and Levenberg–Marquardt optimization. Cheng et al. [26] subsequently proposed a targetless online calibration method based on cross-modal common features, in which deep learning is used to extract shared features and estimate extrinsic parameters online so as to cope with calibration drift during operation. Unlike these studies, which mainly focus on calibration algorithms, this paper assumes that radar-camera extrinsic parameters have been obtained through offline calibration and instead focuses on metric recovery of monocular depth using 4D radar static points under known calibration conditions. It should be noted that calibration error affects the accuracy of radar-depth correspondences; however, the proposed method can suppress its influence to some extent through robust fitting and temporal smoothing. In fusion detection, early methods such as CenterFusion [14] mainly adopted frustum-association strategies, whereas more recent methods such as RCFusion [15] and SGDet3D [16] have shifted toward feature-level fusion in BEV space to improve detection performance. In the context of multi-object tracking, recent studies have begun to pay more attention to the complementary roles of radar and vision in state prediction, data association, and trajectory maintenance. Cheng et al. [22] proposed a fusion MOT method based on Bi-LSTM and appearance-feature modeling, and improved robustness under sensor-failure scenarios through a multi-output mechanism. Cheng et al. [27] further improved association accuracy by combining online extrinsic updates with category-consistency checking. Tang et al. [18] designed a modular tracking framework for the fusion of 4D millimeter-wave radar and monocular vision, and alleviated cross-modal association ambiguity by performing detection matching in the camera domain. These studies indicate that performance gains in fusion-based MOT depend not only on single-frame detection quality but also on stable geometric observations, reliable data association, and trajectory continuity in complex scenes. Overall, existing methods mainly improve fusion tracking through end-to-end modeling, online calibration, or cross-modal matching strategies, whereas this paper focuses on robustly fitting monocular relative depth in the disparity domain using 4D radar static points and on obtaining more stable metric depth observations through temporal smoothing, thereby providing a more reliable 3D geometric foundation for subsequent detection-layer fusion and tracking-layer association.

## 3. Methodology

### 3.1. System Overview

**Figure 1:**
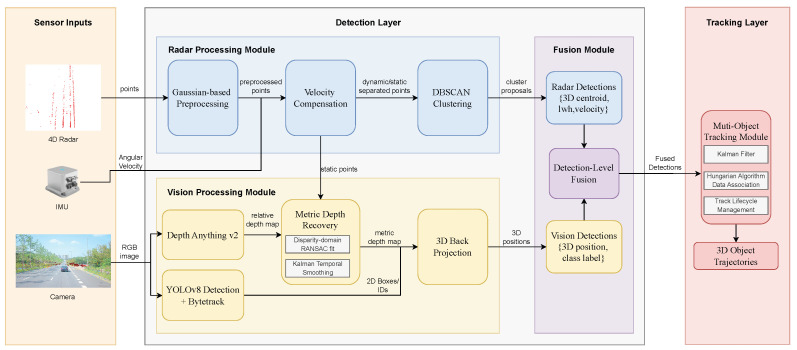
Overall system architecture. The sensor input layer includes 4D millimeter-wave radar, camera, and IMU; the detection layer is divided into radar processing module and visual processing module, generating radar detections and visual detections, respectively; the fusion module implements detection-layer fusion; the tracking layer employs Kalman filtering and Hungarian algorithm for multi-object tracking, ultimately outputting 3D object trajectories.

The overall architecture of the proposed 4D millimeter-wave radar and camera fusion multi-object tracking system is shown in Figure 1. The system follows a hierarchical processing pipeline that can be divided into three layers: sensor input, detection, and tracking.

The data processing flow of the system is summarized as follows.

First, the sensor input layer receives 4D millimeter-wave radar point clouds, front-view camera images, and IMU data. The radar provides object measurements such as 3D position and radial velocity, the camera provides texture and semantic cues, and the IMU provides angular velocity for subsequent velocity compensation.

Second, the detection layer contains two parallel branches. The radar branch suppresses elevation-angle noise through Gaussian distribution-based preprocessing, combines IMU information for velocity compensation to distinguish static and dynamic points, and employs DBSCAN clustering to generate radar detection boxes. The visual branch uses YOLOv8 for 2D object detection and ByteTrack for tracking, applies Depth Anything V2 to estimate monocular relative depth, restores metric depth through RANSAC-based robust fitting in the disparity domain using radar static points, and, finally, back-projects the 2D detections into 3D space.

Next, the fusion module maps radar detections and visual 3D localizations into a unified coordinate system for spatial association, combining visual semantic information with radar velocity cues to produce more complete fusion detections.

Finally, the tracking layer employs a linear Kalman filter for object-state prediction and update, uses the Hungarian algorithm for optimal trajectory-to-detection matching, and handles object appearance, disappearance, and occlusion through trajectory management mechanisms, ultimately outputting object trajectories containing 3D position, velocity, category, and ID.

Through this hierarchical architecture, the system fully exploits the complementary advantages of radar and vision to achieve robust 3D multi-object tracking. The following sections describe the key technical details of each module.

### 3.2. 4D Millimeter-Wave Radar Point Cloud Processing

This section details the processing pipeline for 4D millimeter-wave radar point clouds, including three key steps: Gaussian distribution-based data preprocessing, IMU-based velocity compensation, and DBSCAN density clustering.

#### 3.2.1. Gaussian Distribution-Based Data Preprocessing Method

Compared with conventional 3D radar, 4D millimeter-wave radar provides elevation-angle measurements. However, owing to the limited size of the vertical antenna array, the elevation measurements are considerably noisier, leading to pronounced vertical dispersion of the point cloud and even numerous outliers below the ground surface, which in turn degrade subsequent clustering and object detection accuracy. To mitigate this issue, this paper employs the Gaussian distribution-based point cloud preprocessing strategy proposed in [5], suppressing geometric distortion caused by elevation angle noise through adaptive angle compression.

This method uses Gaussian distribution as a reference model, measuring the degree of deviation of elevation angle distribution from normality through skewness and kurtosis, and adaptively selects the compression reference angle $\theta_{m}$ (mean or median) accordingly. The specific steps are as follows.

Step 1: Elevation angle (divergence angle) calculation. For each point $p_{i}=\left(x_{i},y_{i},z_{i}\right)$ in the radar point cloud, calculate its elevation angle (divergence angle):

(1) $$\theta_{i}=\arctan\left(\frac{z_{i}}{\sqrt{x_{i}^{2}+y_{i}^{2}}}\right)$$

Step 2: Statistical characteristic analysis. Calculate the mean μ and standard deviation σ of the elevation angle sequence $\left\{\theta_{i}\right\}$, and compute the skewness $g_{1}$ and excess kurtosis $g_{2}$:

(2) $$g_{1}=E\left[{\left(\frac{\theta_{i}-\mu }{\sigma }\right)}^{3}\right],\;g_{2}=E\left[{\left(\frac{\theta_{i}-\mu }{\sigma }\right)}^{4}\right]-3$$

where E[·] denotes the sample mean operation over all points in the frame. For a standard normal distribution, $g_{1}=0$ and $g_{2}=0$.

Step 3: Distribution type determination and reference angle selection. When $|g_{1}|\;<\;1$ and $|g_{2}|\;<\;1$, the frame’s elevation angles are considered to approximately follow a Gaussian distribution, and the reference angle is set to the mean $\theta_{m}\;=\;\mu$; otherwise, skewness or heavy-tailed outliers are considered present, and the reference angle is set to the median $\theta_{m}=\mathrm{median}\left(\left\{\theta_{i}\right\}\right)$ to enhance robustness against anomalous points.

Step 4: Adaptive angle compression. Setting the target compression angle $\theta_{t}$ (this paper uses $2^{\circ }$), perform angle compression on the point cloud in the *x*-*z* plane, updating coordinates as:

(3) $$\left\{\begin{array}{c} x_{i}^{\prime }=\cos\;\left(\frac{2\theta_{t}}{\theta_{m}}\cdot \mathrm{atan}2\left(z_{i},x_{i}\right)\right)\cdot \sqrt{x_{i}^{2}+z_{i}^{2}}\\ z_{i}^{\prime }=\sin\;\left(\frac{2\theta_{t}}{\theta_{m}}\cdot \mathrm{atan}2\left(z_{i},x_{i}\right)\right)\cdot \sqrt{x_{i}^{2}+z_{i}^{2}}\\ y_{i}^{\prime }=y_{i} \end{array}\right.$$

where $\left(x_{i},z_{i}\right)$ and $\left(x_{i}^{\prime },z_{i}^{\prime }\right)$ are the coordinates before and after compression respectively, and $\theta_{m}$ is the reference angle obtained in Step 3. This transformation scales the vertical slope angle in the *x*-*z* plane through the ratio $2\theta_{t}/\theta_{m}$, compressing the height direction divergence to a range comparable to $\theta_{t}$, thereby reducing outliers below the ground caused by elevation angle noise.

**Figure 2:**
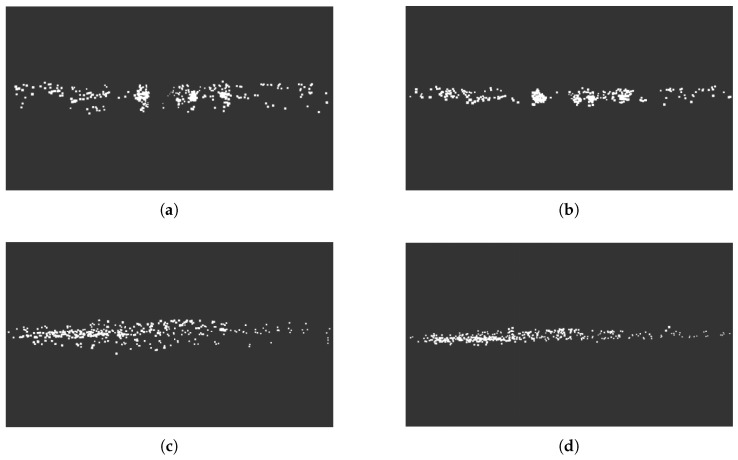
Comparison of Gaussian distribution-based data preprocessing effects. (**a**) Front view of original point cloud; (**b**) front view after preprocessing; (**c**) side view of original point cloud; (**d**) side view after preprocessing.

Figure 2 shows the comparison of Gaussian distribution-based data preprocessing effects. From the front views (a,b), it can be observed that the original point cloud exhibits obvious divergence in the height direction, with some points distributed below the ground (negative height region); after preprocessing, the point cloud distribution in the vertical direction becomes more concentrated, and outliers below the ground are effectively suppressed. From the side views (c,d), the large elevation angle divergence of the original point cloud can be more clearly observed, while the height distribution of the preprocessed point cloud is compressed to a reasonable range, providing more reliable metric anchors for subsequent radar-camera depth fusion.

#### 3.2.2. Velocity Compensation

The 4D millimeter-wave radar directly measures the radial relative velocity of objects with respect to the radar, rather than the absolute velocity of objects relative to the ground. Under ego-motion, velocity compensation is required to recover the true motion state of the object.

This paper employs a rigid body kinematics model for ego-vehicle motion compensation [28]. In typical road scenarios, the vertical velocity of ground vehicles can be neglected, and the ego-vehicle velocity in the body coordinate system is approximately $v_{vehicle}={\left[v_{x},v_{y},0\right]}^{T}$, where $v_{x}$ and $v_{y}$ are the longitudinal and lateral velocities respectively. The angular velocity measured by the IMU is $\omega ={\left[\omega_{x},\omega_{y},\omega_{z}\right]}^{T}$, where $\omega_{x}$, $\omega_{y}$, and $\omega_{z}$ are the roll, pitch, and yaw angular velocities respectively. The offset of the radar installation position relative to the body coordinate system origin is $t_{rc}$, and the velocity at the radar position is:

(4) $$v_{radar}=v_{vehicle}+\omega \times t_{rc}$$

The radar point $p_{r}$ is transformed to the body coordinate system through extrinsic parameters to obtain $p_{V}$. For the object point $p_{V}$ in the body coordinate system, define the line-of-sight unit vector from the radar origin to the object:

(5) $$u=\frac{p_{V}-t_{rc}}{∥p_{V}-t_{rc}∥}$$

The ego-vehicle velocity component in the line-of-sight direction is:

(6) $$v_{ego}=v_{radar}\cdot u$$

Finally, the absolute radial velocity of the object relative to the ground is:

(7) $$v_{r}=v_{doppler}+v_{ego}$$

where $v_{doppler}$ is the Doppler velocity directly measured by the radar.

Based on the compensated absolute radial velocity $v_{r}$, a velocity threshold of $v_{th}=1$ m/s is used to classify points as dynamic or static. Points with $|v_{r}|\;\geq v_{th}$ are labeled as dynamic, whereas points with $|v_{r}|\;<v_{th}$ are labeled as static. This classification serves as the basis for the subsequent clustering strategy.

#### 3.2.3. DBSCAN Clustering

After Gaussian distribution-based data preprocessing and velocity compensation, this paper employs the DBSCAN (Density-Based Spatial Clustering of Applications with Noise) algorithm [29] to cluster radar point clouds and generate object detection boxes. DBSCAN is a density-based clustering algorithm that does not require pre-specifying the number of clusters and can effectively identify clusters of arbitrary shapes while rejecting noise points, making it particularly suitable for processing non-uniformly distributed radar point cloud data.

Traditional DBSCAN only considers spatial distance, but the Doppler velocity information provided by 4D radar offers important cues for distinguishing different moving objects. This paper designs a spatial-velocity joint distance metric, defining the neighborhood criterion between two points $p_{i}$ and $p_{j}$ as:

(8) $$\mathrm{isNeighbor}\left(p_{i},p_{j}\right)=\left\{\begin{array}{cc} \mathrm{True}, & d_{spatial}\left(p_{i},p_{j}\right)\leq \epsilon_{spatial}\;\mathrm{and}\;\left|v_{i}-v_{j}\right|\leq \epsilon_{velocity}\\ \mathrm{False}, & \mathrm{otherwise} \end{array}\right.$$

where $d_{spatial}\left(p_{i},p_{j}\right)=\sqrt{{\left(x_{i}-x_{j}\right)}^{2}+{\left(y_{i}-y_{j}\right)}^{2}+{\left(z_{i}-z_{j}\right)}^{2}}$ is the spatial Euclidean distance, $v_{i}$ and $v_{j}$ are the compensated radial velocities, and $\epsilon_{spatial}$ and $\epsilon_{velocity}$ are the spatial and velocity neighborhood thresholds respectively (this paper sets $\epsilon_{spatial}=2.5$ m, $\epsilon_{velocity}=1.5$ m/s). This joint criterion ensures that points within the same cluster are not only spatially close but also have consistent motion states, effectively avoiding clustering spatially overlapping objects with opposite motion directions into one class. The condition for point $p_{i}$ to be classified as a core point is that its neighborhood point count is not less than the minimum point threshold MinPts (set to 2 in this paper):

(9) $$\mathrm{isCorePoint}\left(p_{i}\right)=\mathrm{NeighborCount}\left(p_{i}\right)\geq MinPts$$

where $\mathrm{NeighborCount}(p_{i})$ is the number of neighborhood points satisfying the joint distance metric (including the point itself).

After clustering, the geometric properties of each cluster $C_{k}=\left\{p_{k1},p_{k2},\ldots ,p_{km}\right\}$ are calculated. The centroid position $c_{k}$ of the cluster is determined by the mean coordinates of all points within the cluster:

(10) $$c_{k}=\frac{1}{m}\sum_{i=1}^{m}p_{ki}$$

To describe the geometric dimensions of objects, this paper calculates the Axis-Aligned Bounding Box (AABB) of the cluster. The spans of the bounding box along each coordinate axis are calculated as follows:

(11) $$\begin{array}{cc} l_{k} & =\max_{i}p_{ki,x}-\min_{i}p_{ki,x}\\ w_{k} & =\max_{i}p_{ki,y}-\min_{i}p_{ki,y}\\ h_{k} & =\max_{i}p_{ki,z}-\min_{i}p_{ki,z} \end{array}$$

where $l_{k}$, $w_{k}$, and $h_{k}$ represent the geometric spans of the object in the *x*, *y*, and *z* axis directions, respectively.

Due to the sparsity of 4D radar point clouds, DBSCAN clustering may incorrectly cluster multiple spatially adjacent objects into one large cluster, especially in static scenarios (such as parking lots). To address this issue, this paper designs a recursive cluster splitting algorithm based on geometric constraints. For the initially clustered cluster $C_{k}$, if its bounding box dimensions exceed preset thresholds, recursive splitting is performed:

(12) $$\mathrm{needSplit}\left(C_{k}\right)=\left\{\begin{array}{cc} \mathrm{True}, & (l_{k}>L_{max}\;\mathrm{or}\;w_{k}>W_{max})\;\mathrm{and}\;\mathrm{splitting}\;\mathrm{conditions}\;\mathrm{met}\\ \mathrm{False}, & \mathrm{otherwise} \end{array}\right.$$

where $L_{max}$ and $W_{max}$ are the thresholds for bounding box length and width. For dynamic objects ($|{\bar{v}}_{k}|\;\geq v_{th}$), $L_{max}=4$ m and $W_{max}=3$ m (typical vehicle dimensions) are set; for static objects, $L_{max}=3$ m and $W_{max}=3$ m are set.

The splitting strategy employs midpoint cutting along the longest axis. Let the bounding box center of cluster $C_{k}$ be $\left(x_{mid},y_{mid}\right)$. If $l_{k}>w_{k}$, the cluster is split into two sub-clusters along the *x*-axis midpoint $x_{mid}$:

(13) $$\begin{array}{cc} C_{k}^{\left(1\right)} & =\{p\in C_{k}|p_{x}<x_{mid}\}\\ C_{k}^{\left(2\right)} & =\{p\in C_{k}|p_{x}\geq x_{mid}\} \end{array}$$

Otherwise, splitting is performed along the *y*-axis midpoint $y_{mid}$. This process is performed recursively until all sub-clusters satisfy the size constraints or cannot be further split (sub-cluster point count too small). Recursive splitting effectively avoids misclassifying multiple objects as a single large object, improving detection accuracy in dense scenarios.

Additionally, the average radial velocity ${\bar{v}}_{k}$ of the cluster is calculated as the mean of Doppler velocities of all points:

(14) $${\bar{v}}_{k}=\frac{1}{m}\sum_{i=1}^{m}v_{ki}$$

Finally, the detection result of the *k*-th object can be represented as:

(15) $$O_{k}=\left\{c_{k},{\bar{v}}_{k},l_{k},w_{k},h_{k},{\mathrm{class}}_{k}\right\}$$

where $c_{k}=\left({\bar{x}}_{k},{\bar{y}}_{k},{\bar{z}}_{k}\right)$ is the centroid position of the object, ${\bar{v}}_{k}$ is the average radial velocity, $l_{k}$, $w_{k}$, $h_{k}$ are the length, width, and height of the object bounding box respectively, and ${\mathrm{class}}_{k}$ is the static-dynamic category determined based on the velocity threshold:

(16) $${\mathrm{class}}_{k}=\left\{\begin{array}{cc} \mathrm{Dynamic}, & |{\bar{v}}_{k}|\;\geq v_{th}\\ \mathrm{Static}, & |{\bar{v}}_{k}|\;<v_{th} \end{array}\right.$$

Although the DBSCAN algorithm can effectively handle non-uniformly distributed radar point clouds, it suffers from inter-frame clustering instability when processing static objects. Due to the sparsity and noise characteristics of radar point clouds, the point cloud density and distribution of the same static object (such as roadside guardrails, buildings, parked vehicles, etc.) may fluctuate between adjacent frames, causing inter-frame jumps in DBSCAN clustering results: point clouds originally belonging to the same object may be split into multiple clusters in certain frames, or incorrectly merged with adjacent objects. This clustering instability severely affects the data association performance of subsequent tracking algorithms, leading to frequent object ID switches and trajectory fragmentation.

To address this issue, this paper proposes a temporal consistency clustering strategy guided by historical static boxes. The core idea of this method is to leverage the prior knowledge that static objects remain stationary between adjacent frames, constraining the clustering process of the current frame through the static object detection boxes from the previous frame, thereby ensuring temporal continuity of static object clustering results.

Specifically, let the static object set at time t−1 be $B_{t-1}=\left\{B_{1}^{t-1},B_{2}^{t-1},\ldots ,B_{N}^{t-1}\right\}$, where each object $B_{i}^{t-1}$ is defined by its bounding box parameters $\left\{c_{i}^{t-1},l_{i}^{t-1},w_{i}^{t-1},h_{i}^{t-1}\right\}$. For the input point cloud $P_{t}=\left\{p_{1},p_{2},\ldots ,p_{M}\right\}$ at time *t*, first perform historical box projection and point membership determination:

(17) $$\mathrm{inBox}\left(p_{j},B_{i}^{t-1}\right)=\left\{\begin{array}{cc} \mathrm{True}, & |p_{j,x}-c_{i,x}^{t-1}|\leq \frac{l_{i}^{t-1}}{2}\;\mathrm{and}\;\left|p_{j,y}-c_{i,y}^{t-1}\right|\leq \frac{w_{i}^{t-1}}{2}\\ \; & \mathrm{and}\;|p_{j,z}-c_{i,z}^{t-1}|\leq \frac{h_{i}^{t-1}}{2}+\delta_{z}\\ \mathrm{False}, & \mathrm{otherwise} \end{array}\right.$$

where $\delta_{z}=1$ m is the tolerance in the height direction to compensate for radar altitude measurement uncertainty. For each historical static box $B_{i}^{t-1}$, collect all points in the current frame that fall within the box, forming a candidate cluster ${\tilde{C}}_{i}^{t}=\left\{p_{j}\in P_{t}|\mathrm{inBox}\left(p_{j},B_{i}^{t-1}\right)=\mathrm{True}\right\}$.

To avoid misclassifying noise points as valid objects, a candidate cluster is output as a valid clustering result only when its point count satisfies the minimum point threshold:

(18) $$\mathrm{isValidCluster}\left({\tilde{C}}_{i}^{t}\right)=\left|{\tilde{C}}_{i}^{t}\right|\geq MinPts$$

For candidate clusters satisfying this condition, their geometric properties are directly calculated and output as static object detection results for the current frame, without requiring DBSCAN clustering. This strategy ensures temporal continuity of static objects: as long as there are sufficient radar echo points within the historical box region, the object will be stably detected, avoiding clustering result jumps caused by point cloud density fluctuations.

For the remaining point set $P_{t}^{residual}=P_{t}\setminus {⋃}_{i}{\tilde{C}}_{i}^{t}$ not captured by any historical static box, the standard DBSCAN process is applied for clustering. This hybrid strategy balances temporal stability with new object detection capability: the historical box guidance mechanism ensures continuous tracking of known static objects, while DBSCAN clustering is responsible for discovering newly appearing objects.

**Figure 3:**
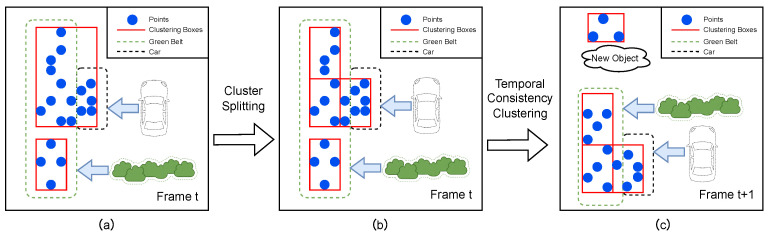
Schematic diagram of radar point cloud clustering optimization pipeline. (**a**) Basic DBSCAN clustering results, where multiple objects are incorrectly aggregated into a large cluster; (**b**) recursive cluster splitting based on geometric constraints, splitting the large cluster into independent objects; (**c**) historical static box guided temporal consistency clustering, where static boxes from time *t* constrain the clustering process at time t+1, while simultaneously detecting newly appearing objects through DBSCAN. The blue arrows indicate the correspondence between physical objects and their associated radar point clusters, while the white block arrows represent the progression of the optimization pipeline.

Figure 3 illustrates the complete processing pipeline of the above clustering optimization methods. As shown in Figure 3a, in the original DBSCAN clustering at time *t*, due to the sparsity of radar point clouds, point clouds from spatially adjacent parked vehicles and roadside green belts are incorrectly aggregated into one large cluster, resulting in over-clustering. After recursive cluster splitting based on geometric constraints (Figure 3b), the oversized cluster is progressively split along its longest axis into independent sub-clusters conforming to single-object dimensions, with each sub-cluster corresponding to an actual physical object. Building upon this, Figure 3c demonstrates the role of historical static box guided clustering at time t+1: the confirmed static object boxes from time *t* (vehicles and green belts) are projected to frame t+1, and point clouds falling within historical boxes that satisfy the MinPts threshold are directly inherited as clustering results for the current frame, ensuring temporal consistency of these static objects; meanwhile, new point clouds not captured by historical boxes are clustered into new objects, ensuring the system’s capability to perceive environmental changes.

### 3.3. Radar-Guided Monocular Metric Depth Recovery

This section details how to achieve metric depth recovery by guiding monocular depth estimation networks with 4D millimeter-wave radar point clouds, addressing the inherent scale ambiguity problem of monocular depth.

#### 3.3.1. Scale-Free Depth Prediction Based on Depth Anything

This paper employs Depth Anything V2 [9] as the base network for monocular depth estimation. This model is based on the Vision Transformer architecture, achieving excellent zero-shot cross-domain generalization capability through self-supervised pretraining on large-scale unlabeled image data combined with knowledge distillation strategies.

Given an input image $I\in R^{H\times W\times 3}$, the network outputs a scale-free depth map $D_{net}\in R^{H\times W}$ at the same resolution as the input. It should be noted that the output of Depth Anything is essentially a relative disparity representation, with its values proportional to the inverse of scene depth, i.e., larger output values indicate closer object distances. The output of such monocular depth estimation networks only reflects the relative near-far relationships of different positions in the scene, rather than true metric depth. To ensure the effectiveness of subsequent disparity-domain linear fitting, this paper directly uses the raw network output during calibration (only resizing to original image resolution), without performing min-max normalization or quantile clipping on $D_{net}$; normalization is only used for visualization display.

As revealed by Ranftl et al. [6], the output $D_{net}$ of monocular depth estimation networks is essentially a relative disparity representation. A linear relationship exists between the inverse of true metric depth $D_{metric}$ and the network output:

(19) $$\frac{1}{D_{metric}}=s\cdot D_{net}+t$$

where *s* is the scale factor and *t* is the offset.

#### 3.3.2. Establishing Radar-Depth Correspondences

To achieve metric calibration of monocular depth, correspondences between radar point clouds and network depth maps need to be established. This paper employs a projection-sampling approach to obtain valid depth correspondence pairs.

After completing static-dynamic filtering and multi-frame static point accumulation at the radar end, 4D millimeter-wave radar points are transformed to the LiDAR coordinate system used for camera projection. Let the point in the LiDAR coordinate system be $p_{L}={\left(x_{L},y_{L},z_{L}\right)}^{T}$, and project it to the image plane using the camera projection matrix $P\in R^{3\times 4}$ (composed of intrinsic parameters K and extrinsic parameters [R|t]):

(20) $$\tilde{p}=P\left[\begin{array}{c} p_{L}\\ 1 \end{array}\right],\;u=\frac{{\tilde{p}}_{x}}{{\tilde{p}}_{z}},\;v=\frac{{\tilde{p}}_{y}}{{\tilde{p}}_{z}}$$

where (u,v) are the projected pixel coordinates. In this paper’s implementation, the calibrated P is directly used to project radar points, with boundary validity checks performed on projected points.

For valid projected points (u,v), bilinear interpolation is used to sample the corresponding prediction $d_{i}^{net}$ from the network output depth map $D_{net}$, thereby obtaining subpixel-accurate depth estimates and reducing quantization noise. For samples near the image boundary, nearest-neighbor interpolation is used instead to avoid out-of-bounds access. In particular, zero padding is avoided, because a zero value corresponds to infinite distance in the disparity domain and would severely corrupt the subsequent RANSAC fitting.

Considering the sparsity of single-frame radar point clouds, this paper employs a multi-frame accumulation strategy to increase the number of valid correspondence points. For static points (compensated radial velocity below threshold), point clouds from the most recent *N* frames are accumulated (in this paper’s implementation, N=3). Since historical frame point cloud coordinates have changed with ego-vehicle motion, recalculation based on global coordinates and current pose is required. Let the global coordinates of a historical static point be $p_{\mathrm{global}}$, and the current frame’s body pose be (t,θ), then the coordinates of this point in the current body coordinate system are:

(21) $$p_{\mathrm{car}}=R_{z}\left(-\theta \right)\cdot \left(p_{\mathrm{global}}-t\right)$$

where $R_{z}\left(-\theta \right)$ is the rotation matrix rotating −θ around the *z*-axis. The final coordinates are obtained through the transformation matrix from body to sensor reference coordinate system. This strategy significantly increases the number of correspondence points available for calibration while ensuring point cloud timeliness.

Through the above processing, a set of valid depth correspondence pairs ${\left\{\left(d_{i}^{net},d_{i}^{ref}\right)\right\}}_{i=1}^{M}$ is obtained. Here $d_{i}^{net}$ is the network output value; the reference depth $d_{i}^{ref}$ is taken as the *Z* coordinate of the radar point in the camera coordinate system: let $p_{C}=T_{LC}\left[\begin{array}{c} p_{L}\\ 1 \end{array}\right]={\left(X_{c},Y_{c},Z_{c},1\right)}^{T}$, then $d_{i}^{ref}=Z_{c}$.

#### 3.3.3. Disparity-Domain RANSAC Depth Calibration

Based on the depth correspondence pairs established in the previous section, this section introduces how to recover metric depth through robust fitting. According to the previous analysis, the network output exhibits a linear relationship with true depth in the disparity domain, so this paper performs parameter fitting in the disparity domain rather than the depth domain.

First, convert true depth values to disparity values:

(22) $${\tilde{d}}_{i}^{ref}=\frac{1}{d_{i}^{ref}}$$

In the disparity domain, the network output $d_{i}^{net}$ and true disparity ${\tilde{d}}_{i}^{ref}$ satisfy a linear relationship:

(23) $${\tilde{d}}_{i}^{ref}=s\cdot d_{i}^{net}+t$$

The choice of inlier determination threshold significantly affects fitting results. This paper employs an adaptive threshold calculation method based on Median Absolute Deviation (MAD). First, initial fitting is performed using least squares, residuals $\left\{r_{i}\right\}$ for all points are calculated, and then the adaptive threshold is computed:

(24) $$\tau =\mathrm{median}(|r_{i}\left|\right)+k\cdot \mathrm{MAD}(|r_{i}\left|\right)$$

where $\mathrm{MAD}(|r_{i}\left|\right)=\mathrm{median}\left(\left|\right|r_{i}\left|-\mathrm{median}(\right|r_{i}\left|)\right|\right)$ is the median absolute deviation, and *k* is the scale coefficient (set to k=2.5 in this paper’s implementation). The MAD method is more robust to outliers compared to standard deviation and can adaptively adjust the threshold according to the noise level of current frame data.

Since radar point clouds may contain noise and outliers, direct least squares fitting is susceptible to anomalous values. This paper employs the RANSAC algorithm for robust parameter estimation: randomly sample the minimum number of points (2 points) from the correspondence set to fit a linear model, use the above adaptive threshold τ to calculate residuals for all points and count the number of inliers, and retain the model with the most inliers after iteration. To ensure physical reasonableness, constraints are imposed on candidate models: scale factor s∈[0.001,0.1], offset |t|≤0.5.

Using the inlier set I determined by RANSAC, least squares is used to refit parameters (s,t) for more accurate estimation:

(25) $$\left(s^{*},t^{*}\right)=\arg\min_{s,t}\sum_{i\in I}{\left({\tilde{d}}_{i}^{ref}-s\cdot d_{i}^{net}-t\right)}^{2}$$

To avoid jitter in single-frame fitting parameters, this paper employs a Kalman filter for temporal smoothing of scale factor and offset. The Kalman filter can adaptively adjust filter gain according to observation quality and provides uncertainty quantification of state estimates.

Define the state vector $x_{k}={\left[s_{k},t_{k}\right]}^{T}$, containing the scale factor and offset in the disparity domain. Assuming parameters change slowly between adjacent frames, an identity state transition model is adopted:

(26) $$x_{k}=Fx_{k-1}+w_{k-1},\;F=I_{2\times 2}$$

where $w_{k-1}∼N\left(0,Q\right)$ is the process noise, and $Q=\mathrm{diag}(q_{s}^{2},q_{t}^{2})$ is the process noise covariance matrix.

The observation equation is:

(27) $$z_{k}=Hx_{k}+v_{k},\;H=I_{2\times 2}$$

where $z_{k}={\left[s^{*},t^{*}\right]}^{T}$ are the parameters fitted by RANSAC, and $v_{k}∼N\left(0,R\right)$ is the observation noise.

The Kalman filter prediction step is:

(28) $${\hat{x}}_{k|k-1}=F{\hat{x}}_{k-1|k-1},\;P_{k|k-1}=FP_{k-1|k-1}F^{T}+Q$$

The update step is:

(29) $$K_{k}=P_{k|k-1}H^{T}{\left(HP_{k|k-1}H^{T}+R\right)}^{-1}$$

(30) $${\hat{x}}_{k|k}={\hat{x}}_{k|k-1}+K_{k}\left(z_{k}-H{\hat{x}}_{k|k-1}\right)$$

(31) $$P_{k|k}=\left(I-K_{k}H\right)P_{k|k-1}$$

To fully utilize RANSAC fitting quality information, this paper estimates observation noise covariance based on linear regression theory. Let the design matrix for disparity-domain fitting be $X\in R^{n\times 2}$, where *n* is the number of inliers, then the covariance of parameter estimates is:

(32) $$\mathrm{Cov}\left(\hat{\beta }\right)=\sigma^{2}{\left(X^{T}X\right)}^{-1}$$

where σ is the residual standard deviation, estimated robustly using MAD (Median Absolute Deviation):

(33) $$\hat{\sigma }=1.4826\cdot \mathrm{median}\left(|r_{i}-\mathrm{median}\left(r_{i}\right)|\right)$$

The coefficient $1.4826=1/\Phi^{-1}\left(0.75\right)$ is the correction factor under normal distribution, ensuring MAD estimation maintains consistency with standard deviation under Gaussian noise. The observation noise covariance matrix is set as:

(34) $$R={\hat{\sigma }}^{2}{\left(X^{T}X\right)}^{-1}$$

This method, compared to simple scalar noise models, can correctly reflect the uncertainty and correlation of scale factor and offset estimates.

To further suppress the influence of outlier observations, this paper employs Normalized Innovation Squared (NIS) for gating tests:

(35) $$\mathrm{NIS}=y_{k}^{T}S_{k}^{-1}y_{k},\;y_{k}=z_{k}-H{\hat{x}}_{k|k-1},\;S_{k}=HP_{k|k-1}H^{T}+R$$

When $\mathrm{NIS}>\chi_{2,0.99}^{2}\approx 9.21$, the current observation is considered an outlier, and only the prediction step is executed while skipping the update step. In this case, the covariance $P_{k|k}=P_{k|k-1}$ increases, reflecting the rise in state estimation uncertainty.

Using the Kalman filter smoothed parameters $\left({\hat{s}}_{k},{\hat{t}}_{k}\right)$, the network output is converted to a metric depth map:

(36) $$D_{metric}\left(u,v\right)=\frac{1}{|{\hat{s}}_{k}\cdot D_{net}\left(u,v\right)+{\hat{t}}_{k}|+\epsilon }$$

where ϵ is a small constant to prevent division by zero (this paper uses $\epsilon =10^{-3}$), and the absolute value is taken to handle possible negative disparity values, ensuring depth values are always positive.

Compared to direct linear fitting in the depth domain, disparity-domain fitting has the following advantages: (1) it conforms to the intrinsic characteristics of monocular depth network outputs, with better matching between fitting model and data distribution; (2) distant objects have smaller values in the disparity domain and do not dominate the fitting process, avoiding the problem of amplified distant errors in depth-domain fitting; (3) the linear relationship in the disparity domain makes RANSAC inlier determination more accurate, improving robustness against radar noise and outliers.

#### 3.3.4. Depth Back-Projection and Object Localization

Based on the metric depth map $D_{metric}$ obtained in the previous section, this section introduces how to convert 2D image information to 3D spatial positions, achieving precise object localization. The overall process includes: 2D object detection, depth sampling, pixel back-projection, multi-level coordinate system transformation, and optional temporal smoothing.

To obtain image positions of objects of interest in the scene, this paper employs the YOLOv8 object detection network to process input images, combined with the ByteTrack [30] algorithm for 2D multi-object tracking. YOLOv8 is an efficient single-stage object detector that can output 2D bounding boxes B=(x,y,w,h) and category labels in real-time; ByteTrack maintains stable object IDs in occlusion scenarios by associating high and low confidence detection boxes. Detection and tracking results provide object-level regions of interest and temporal association information for subsequent depth sampling and 3D localization.

For each detected 2D bounding box, a representative depth value for that object needs to be obtained from the metric depth map. Considering that object detection boxes may contain background regions and local noise in depth estimation, this paper employs a bottom region sampling strategy. Specifically, first, calculate the rectangular range of the bottom 1/3 region of the detection box:

(37) $$R_{bottom}=\left\{\left(u,v\right)|x\leq u<x+w,\;y+\frac{2h}{3}\leq v<y+h\right\}$$

Then, extract all depth values within this region, filter invalid values (depth < 0.1 m or >200 m or non-finite values), and calculate the median as the object depth $d_{obj}$. The theoretical basis for selecting the bottom region is that this area is closer to the contact point between the object and the ground, where depth estimation is relatively more accurate; using the median instead of the mean effectively suppresses the influence of outliers, improving robustness.

To ensure consistency between depth sampling position and back-projection pixel coordinates, this paper uses the center point of the bottom 1/3 region as the back-projection reference point:

(38) $$u_{c}=x+\frac{w}{2},\;v_{c}=y+\frac{5h}{6}$$

Combined with the object depth $d_{obj}$, back-projection is performed using the camera intrinsic matrix K to obtain the 3D position of the object in the camera coordinate system:

(39) $$p_{C}=d_{obj}\cdot K^{-1}\left[\begin{array}{c} u_{c}\\ v_{c}\\ 1 \end{array}\right]=\left[\begin{array}{c} \left(u_{c}-c_{x}\right)\cdot d_{obj}/f_{x}\\ \left(v_{c}-c_{y}\right)\cdot d_{obj}/f_{y}\\ d_{obj} \end{array}\right]$$

where $\left(f_{x},f_{y}\right)$ are the camera focal lengths and $\left(c_{x},c_{y}\right)$ are the principal point coordinates.

Because the system involves multiple coordinate frames, a sequence of coordinate transformations is required to obtain the final object position in the vehicle coordinate system. The coordinate transformation chain is as follows:

(40) $$\mathrm{Camera}\;\mathrm{coordinate}\;\mathrm{system}\overset{T_{C\to L}}{\to }\mathrm{LiDAR}\;\mathrm{coordinate}\;\mathrm{system}\overset{T_{L\to V}}{\to }\mathrm{Body}\;\mathrm{coordinate}\;\mathrm{system}$$

First, transform the 3D position in the camera coordinate system to the LiDAR coordinate system. Let $T_{L\to C}=\left[R_{LC}|t_{LC}\right]$ be the extrinsic transformation matrix from LiDAR to camera (obtained through calibration), then the inverse transformation from camera to LiDAR is:

(41) $$p_{L}=R_{LC}^{T}\left(p_{C}-t_{LC}\right)$$

Then, transform the position in the LiDAR coordinate system to the body coordinate system:

(42) $$p_{V}=T_{L\to V}\cdot \left[\begin{array}{c} p_{L}\\ 1 \end{array}\right]$$

where $T_{L\to V}$ is the rigid body transformation matrix from LiDAR to body. The resulting $p_{V}={\left(x_{V},y_{V},z_{V}\right)}^{T}$ is the 3D position of the object in the body coordinate system.

Due to inter-frame jitter in monocular depth estimation, this paper uses the One-Euro filter [31] for temporal smoothing of object positions. The One-Euro filter is an adaptive low-pass filter whose cutoff frequency dynamically adjusts according to signal change rate: when objects move slowly, a lower cutoff frequency is used for sufficient smoothing; when objects move quickly, the cutoff frequency is increased to reduce lag.

Based on the object 3D center position $p_{V}$ (body coordinate system), combined with category-based prior dimensions, complete 3D bounding boxes are constructed. This paper employs a category-based standard dimension database, presetting typical length, width, and height dimensions for different object categories (such as vehicles, pedestrians, etc.). Finally, the 3D bounding box is represented by center position $p_{V}$ and dimensions (l,w,h).

Through the above process, the system achieves complete conversion from 2D image detection to 3D spatial localization. Compared to pure radar point cloud clustering methods, depth estimation-based 3D localization can provide independent depth estimates for each visual detection object, avoiding object miss-detection caused by sparse radar point clouds, while maintaining one-to-one correspondence with visual detection boxes, facilitating subsequent multi-object tracking.

### 3.4. Multi-Sensor Fusion and Object Tracking

This section introduces how to fuse radar detection results with visual depth localization results and achieve continuous object tracking through multi-object tracking algorithms.

#### 3.4.1. Detection-Layer Fusion

At the detection layer, this paper performs spatial association between radar clustered objects and visual depth-localized objects, achieving complementary fusion of heterogeneous sensor information.

After DBSCAN clustering, each radar object is represented by its centroid position $p_{\mathrm{radar}}={\left(x_{r},y_{r},z_{r}\right)}^{T}$, bounding box dimensions $\left(l_{r},w_{r},h_{r}\right)$, average radial velocity $v_{r}$, and object category (dynamic/static) attributes. After depth back-projection, each visual object is represented by its 3D center position $p_{\mathrm{vision}}={\left(x_{v},y_{v},z_{v}\right)}^{T}$, category label *c*, detection confidence *s*, and 2D bounding box B attributes.

Radar objects and visual objects are matched by spatial distance in the unified body coordinate system. For visual object *i* and radar object *j*, calculate their Euclidean distance on the horizontal plane:

(43) $$d_{ij}=\sqrt{{\left(x_{v}^{i}-x_{r}^{j}\right)}^{2}+{\left(y_{v}^{i}-y_{r}^{j}\right)}^{2}}$$

Considering the motion characteristic differences between dynamic and static objects, this paper employs a hierarchical matching strategy: first search for the nearest neighbor among radar dynamic objects (VEHICLE class), and if the distance is less than the dynamic threshold $\tau_{dynamic}$, matching is successful; if no match is found, search for the nearest neighbor among all radar objects, using the static threshold $\tau_{static}$ for determination. This strategy provides larger matching tolerance for high-speed moving objects while maintaining matching accuracy in static scenarios.

For successfully associated object pairs, the following fusion strategy is adopted:

- Position information: Radar measurements are retained, as they have direct ranging capability and higher ranging accuracy;
- Velocity information: Radar-measured radial velocity is adopted, as it has direct Doppler measurement capability;
- Category information: Visual detection category labels are adopted, as they have richer semantic recognition capability.

For unassociated radar objects and visual objects, both are retained as independent detection results to participate in subsequent tracking. This strategy ensures the complementarity of both sensors: vision provides dense semantic detection, while radar provides reliable position and velocity measurements as well as all-weather perception capability.

#### 3.4.2. State Estimation Based on Linear Kalman Filter

This paper employs a linear Kalman filter for recursive estimation of object states, using the Constant Velocity (CV) motion model.

The state vector is defined as:

(44) $$x={\left[x,y,v_{x},v_{y}\right]}^{T}$$

where (x,y) is the object position in the global coordinate system, and $\left(v_{x},v_{y}\right)$ are velocity components.

Given time interval Δt, the state transition equation of the CV model is:

(45) $$x_{k|k-1}=Fx_{k-1|k-1},\;F=\left[\begin{array}{cccc} 1 & 0 & \Delta t & 0\\ 0 & 1 & 0 & \Delta t\\ 0 & 0 & 1 & 0\\ 0 & 0 & 0 & 1 \end{array}\right]$$

Covariance prediction is:

(46) $$P_{k|k-1}=FP_{k-1|k-1}F^{T}+Q$$

where Q is the process noise covariance matrix, used to model object motion uncertainty.

The observation vector is the position measurement of the object $z_{k}={\left[x_{m},y_{m}\right]}^{T}$, and the observation matrix is:

(47) $$H=\left[\begin{array}{cccc} 1 & 0 & 0 & 0\\ 0 & 1 & 0 & 0 \end{array}\right]$$

When a new observation $z_{k}$ is obtained, calculate the Kalman gain:

(48) $$K_{k}=P_{k|k-1}H^{T}{\left(HP_{k|k-1}H^{T}+R\right)}^{-1}$$

where R is the observation noise covariance matrix.

State and covariance updates are:

(49) $$x_{k|k}=x_{k|k-1}+K_{k}\left(z_{k}-Hx_{k|k-1}\right)$$

(50) $$P_{k|k}=\left(I-K_{k}H\right)P_{k|k-1}$$

The advantages of the linear Kalman filter lie in high computational efficiency and simple implementation, with good adaptability to the approximately constant velocity motion of most objects in urban road scenarios.

#### 3.4.3. Data Association Based on Hungarian Algorithm

At the tracking layer, this paper employs a Hungarian algorithm-based data association method to optimally match current frame fusion detection results with historical trajectories.

Let the current frame have *M* detected objects and the historical trajectory library have *N* active trajectories. For trajectory *i* and detection *j*, construct the cost function:

(51) $$C_{ij}=d_{pos}\left(p_{i}^{pred},p_{j}^{det}\right)+\lambda_{v}\cdot \left|v_{i}^{pred}-v_{j}^{det}\right|$$

where $p_{i}^{pred}$ is the Kalman filter predicted position of trajectory *i*, $p_{j}^{det}$ is the observed position of detection *j*, $d_{pos}\left(\cdot ,\cdot \right)$ is the position Euclidean distance, $v_{i}^{pred}$ and $v_{j}^{det}$ are the radial components of predicted velocity and observed velocity respectively, and $\lambda_{v}$ is the velocity weight coefficient.

Based on the cost matrix $C\in R^{N\times M}$, the Hungarian algorithm is used to solve the optimal bipartite graph matching problem:

(52) $$\min_{\pi }\sum_{i=1}^{N}C_{i,\pi (i)}$$

where π is the matching mapping function. To avoid erroneous associations, a cost threshold $\tau_{c}$ is set; when $C_{ij}>\tau_{c}$, trajectory *i* and detection *j* are considered non-associable.

Based on the Hungarian algorithm matching results, trajectories and detections are classified for processing: for successfully matched trajectory-detection pairs, detection observations are updated to corresponding trajectories through Kalman filtering, and the unmatched count is reset; for unmatched trajectories, the unmatched count is incremented, and if consecutive unmatched frames exceed the threshold, the trajectory is terminated; for unmatched detections, new trajectories are initialized and enter candidate status.

#### 3.4.4. Trajectory Management

To ensure the stability and real-time performance of the tracking system, this paper designs a complete trajectory lifecycle management mechanism.

When a detected object cannot be associated with any existing trajectory, a new trajectory is created for it. New trajectories are initially in “candidate” status and need to be successfully matched for consecutive multiple frames before transitioning to “confirmed” status, to avoid short-lived trajectories generated by false alarm objects.

When a trajectory fails to match any detected object for consecutive multiple frames, the object is considered to have left the field of view or been occluded, and the trajectory transitions to “historical” status. Historical trajectories can still be reactivated within a certain time window to handle object reappearance after brief occlusion.

Post-processing is performed on each trajectory: trajectory-level velocity and heading angle are calculated through historical frame averaging, radial velocity correction is performed for initial-stage trajectories, and trajectory category is determined based on historical frame statistics and visual information.

Through the above multi-level fusion and tracking strategies, the system can fully exploit the complementary advantages of radar and visual sensors: vision provides rich semantic information and dense object detection, while radar provides accurate velocity measurements and all-weather perception capability. The linear Kalman filter achieves efficient estimation of object states, the Hungarian algorithm ensures global optimality of data association, and the trajectory management mechanism ensures long-term stable operation of the tracking system.

## 4. Experiments and Analysis

This section provides experimental validation and analysis of the proposed 4D millimeter-wave radar and camera fusion multi-object tracking algorithm.

### 4.1. Experimental Setup

#### 4.1.1. Experimental Platform and Sensor Configuration

This paper conducts experimental validation on a self-collected dataset. The experimental platform is equipped with a Sensing SG2IMX390CH60N industrial camera (SZ Sensing TECH Co., Ltd., Shenzhen, China), a Continental ARS548 4D imaging millimeter-wave radar (Continental Engineering Services GmbH, Frankfurt am Main, Germany), and an XW-GI7660 inertial navigation system (Beijing Xingwang Yuda Technology Co., Ltd., Beijing, China). The camera resolution is 1920×1080 pixels with an acquisition frequency of 10 Hz; three millimeter-wave radars are configured, installed at the front, left side, and right side of the vehicle respectively, with maximum detection range up to 300 m, velocity resolution of 0.1 m/s, and acquisition frequency of 20 Hz; the IMU acquisition frequency is 125 Hz, used to provide vehicle attitude and angular velocity information for radar point cloud velocity compensation.

#### 4.1.2. Dataset and Annotation

**Figure 4:**
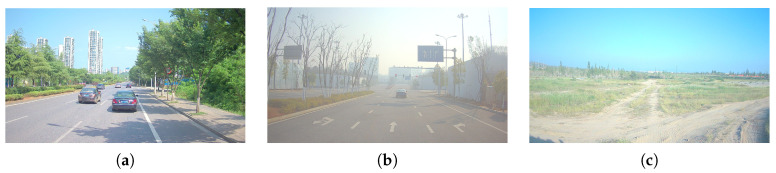
Example images from the three experimental scenes. (**a**) Scene 1: urban road with dense traffic and frequent occlusion; (**b**) Scene 2: urban road with relatively sparse traffic; (**c**) Scene 3: off-road environment with uneven terrain and dust occlusion.

Experimental data were collected in two typical scenarios: urban roads and off-road terrain. The urban setting contains two data sequences (Scene 1 and Scene 2), comprising 2909 and 2699 frames, respectively, and is characterized by narrow roads, dense traffic participants, and frequent occlusions. The off-road setting contains one data sequence (Scene 3) with 1408 frames and is characterized by wide roads, sparse targets, and dust interference. Representative images of the three experimental scenes are shown in Figure 4a–c, corresponding to Scene 1, Scene 2, and Scene 3, respectively.

**Figure 5:**
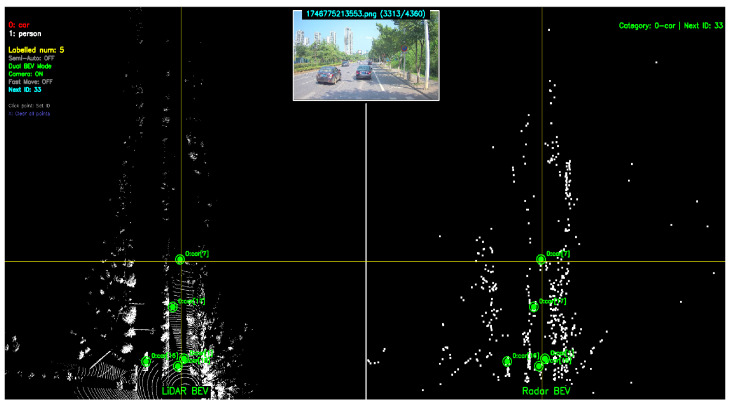
Radar point cloud calibration and annotation tool interface in BEV perspective. The (**left**) panel presents the LiDAR bird’s-eye-view (BEV), the (**right**) panel presents the BEV projection generated from the fused point clouds of the three 4D mmWave radars, and the upper middle panel shows the corresponding camera image. White points denote sensor point clouds, and green circles and labels denote manually annotated objects.

To evaluate tracking algorithm performance, we manually annotated objects in the Bird’s-Eye View (BEV) perspective. The annotation process is as follows: first, camera images, millimeter-wave radar point clouds, and LiDAR point clouds are parsed from ROS bags, with multi-sensor time synchronization based on camera timestamps; then, LiDAR and millimeter-wave radar point clouds are projected to BEV space, and the two radar point clouds are spatially aligned in BEV through a calibration program we developed, as shown in Figure 5; finally, BEV coordinates (x,y) and trajectory IDs are annotated for each object through a visualization annotation tool, serving as ground truth reference for algorithm evaluation. The region of interest for all data sequences is set to 0–80 m in front of the vehicle.

#### 4.1.3. Implementation Details

The proposed algorithm is implemented in C++ within the ROS 1 Noetic framework on Ubuntu 20.04, and the deep learning models are accelerated for inference using TensorRT 8.5.3.1. The object detector is YOLOv8m with an input image resolution of 1920×1080, optimized by TensorRT and executed in FP16 precision. The monocular depth estimation network is Depth Anything V2 with a ViT-Base backbone and an input resolution of 518×518, and it is likewise accelerated by TensorRT in FP16 precision. Millimeter-wave radar point cloud clustering is performed using DBSCAN, with the neighborhood radius ϵ set to 2.5 m and the minimum number of points MinPts set to 2. All experiments are conducted on a laptop equipped with an NVIDIA GeForce RTX 4060 Laptop GPU. The overall system runs at approximately 10 Hz, satisfying real-time requirements.

#### 4.1.4. Evaluation Metrics

This paper employs standard evaluation metrics from the multi-object tracking field to assess algorithm performance. Main metrics include:

(1) FNR (False Negative Rate): Calculated as FNR=FN/GT, where FN is the number of missed detections and GT is the total number of ground truth objects. This metric reflects the proportion of real objects that the system failed to detect.

(2) FPR (False Positive Rate): Calculated as FPR=FP/Pred, where FP is the number of false detections and Pred is the total number of predicted objects. This metric reflects the proportion of false detections produced by the system.

(3) IDSWR (ID Switch Rate): Calculated as IDSWR=IDSW/GT, where IDSW is the number of ID switches. This metric reflects trajectory ID consistency.

(4) MOTA (Multiple Object Tracking Accuracy): Comprehensively considers missed detections, false detections, and ID switches, calculated as:

(53) $$\mathrm{MOTA}=1-\frac{\mathrm{FN}+\mathrm{FP}+\mathrm{IDSW}}{\mathrm{GT}}$$

(5) MOTP (Multiple Object Tracking Precision): Represents the average localization error of successfully matched objects (unit: meters).

#### 4.1.5. Comparison Methods

To comprehensively evaluate the advantages of the proposed fusion framework over single-modality approaches and other fusion strategies, the following comparison methods are considered:

(1) Camera-only method: YOLOv8 detection and Depth Anything depth estimation are used, while radar static points are still used for depth calibration; however, dynamic target information from radar clustering is not involved in fusion tracking.

(2) Radar-only method: Only 4D millimeter-wave radar point clouds are used, and DBSCAN clustering is applied to dynamic points after velocity compensation.

(3) IPM-based fusion method [32]: Inverse perspective mapping is used to project 2D detection boxes into BEV space. Under the flat-ground assumption, object 2D positions are estimated using the camera intrinsics and installation height, then associated with radar measurements via the Hungarian algorithm, and tracked using a multi-object tracking framework built on three Kalman filters.

(4) Point-cloud-projection-based fusion method: Instead of using a depth estimation network, radar point clouds are directly projected onto the image plane and associated with YOLOv8 2D detection boxes. DBSCAN clustering is then performed on radar points falling inside each detection box to obtain the 3D object position. This method is used to verify the benefit of introducing a depth estimation network for fusion accuracy.

(5) Fusion method (Ours): The complete framework combining Depth Anything depth estimation, radar-guided depth recovery with Kalman-filter smoothing, and multi-sensor fusion tracking.

### 4.2. Results and Analysis

#### 4.2.1. Performance Comparison of Different Methods

Table 1 presents the performance comparison of different methods across all scenarios.

Table 1 presents the detailed performance comparison of the proposed fusion framework against camera-only, radar-only, IPM-based fusion [32], and point-cloud-projection-based fusion methods across different scenarios. Overall, the proposed method achieves a clear advantage in MOTA, the core metric of tracking performance, which validates the effectiveness of the framework in overcoming the bottlenecks of single-modality perception.

In terms of comprehensive tracking accuracy (MOTA), the proposed method shows clear superiority. On the overall benchmark, it achieves a MOTA of 77.93%, outperforming the camera-only method by 11.34 percentage points, the radar-only method by 14.79 percentage points, the IPM-based fusion method by 31.47 percentage points, and the point-cloud-projection method by 30.33 percentage points. The advantage is particularly notable in Scene 2, the most challenging congested urban environment, where the proposed fusion method reaches a MOTA of 88.80%, far exceeding the IPM-based fusion method (41.59%) and the point-cloud-projection method (52.18%). These results indicate that multimodal fusion can effectively overcome the perception limitations of individual sensors in complex conditions.

In terms of false negative rate (FNR) and false positive rate (FPR), the fusion strategy achieves complementary benefits. For missed detections, the radar-only method exhibits a high FNR in urban scenes (above 34%), mainly because its DBSCAN-based radar detection pipeline only processes dynamic point clouds and cannot perceive static objects such as parked vehicles, which are common in urban scenes. By introducing dense visual detections, the proposed method reduces the overall FNR from 32.31% for radar-only tracking to 13.47%, effectively compensating for radar’s inability to perceive static targets.

It is worth noting that the point-cloud-projection method attains the lowest FNR overall (8.78%) because it treats all radar points falling inside YOLO detection boxes as valid associations and therefore covers the widest set of detections. However, this comes at the cost of an FPR as high as 31.65%, far above the 8.55% of the proposed method, resulting in a MOTA of only 47.60%. The underlying reasons are twofold. First, vehicle attitude perturbations during driving introduce dynamic extrinsic misalignment between the radar and the camera, causing projected radar points to deviate from the true image regions and thereby leading to missed or erroneous associations. Second, radar points falling inside a detection box do not necessarily originate from the target itself; they may also include ground reflections or background points from farther objects. These interfering points participate in clustering and introduce additional localization errors and false detections. In addition, the point-cloud-projection method exhibits a substantially higher IDSWR (1.39% overall, approximately 2.4 times that of the proposed method), indicating that unstable depth association causes frame-to-frame position jitter and consequently frequent ID switches. These results suggest that relying solely on geometric projection of sparse radar points is insufficient for establishing reliable depth association, whereas introducing a dense depth-estimation network significantly improves fusion accuracy.

In suppressing false detections, the IPM-based fusion method yields an overall FPR of 27.91%, demonstrating the failure of the planar-ground assumption in unstructured scenarios such as off-road terrain and in the presence of non-ground interference. By contrast, the proposed disparity-domain recalibration method avoids the planar assumption and limits the overall FPR to 8.55%, significantly outperforming both the IPM-based and point-cloud-projection-based fusion methods.

In terms of localization precision (MOTP), the proposed fusion method maintains a reasonable localization level while improving recall. The overall MOTP is 1.40 m, which is slightly higher than those of the radar-only method and the point-cloud-projection method (both 1.29 m), as well as the IPM-based fusion method (1.33 m). However, considering the unavoidable residual error introduced by visual depth estimation, this level of precision remains acceptable and is still better than that of the camera-only method (1.41 m). Overall, trading approximately 0.1 m of localization precision for a reduction of nearly 19 percentage points in FNR and an increase of more than 14 percentage points in MOTA is acceptable for practical applications.

**Figure 6:**
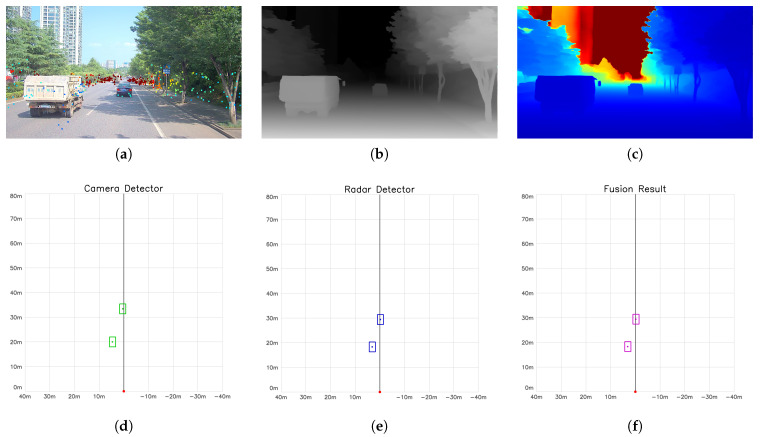
Processing pipeline and detection results in urban scenario. (**a**) Radar point cloud projection; (**b**) raw depth map; (**c**) metric depth map; (**d**) camera detection (BEV); (**e**) radar detection (BEV); (**f**) fusion detection (BEV). All detection results are visualized in the vehicle-body coordinate system, where rectangular boxes denote targets and different colors indicate detections from different methods.

**Figure 7:**
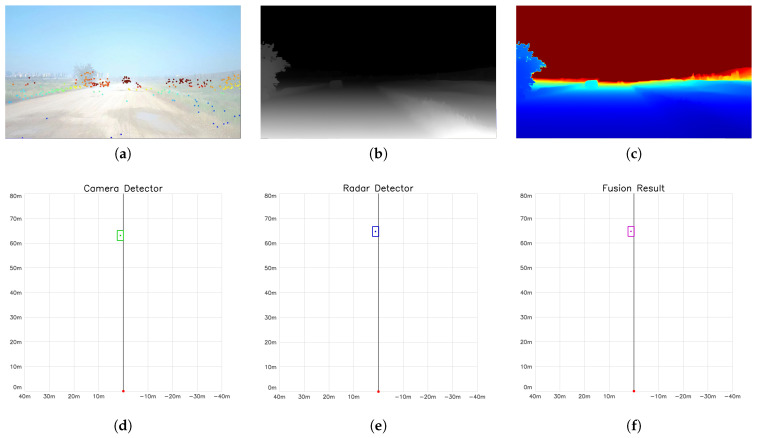
Processing pipeline and detection results in off-road scenario. (**a**) Radar point cloud projection; (**b**) raw depth map; (**c**) metric depth map; (**d**) camera detection (BEV); (**e**) radar detection (BEV); (**f**) fusion detection (BEV). All detection results are visualized in the vehicle-body coordinate system, where rectangular boxes denote targets and different colors indicate detections from different methods.

Figure 6 and Figure 7 present the complete processing pipeline and detection results in urban and off-road scenarios, respectively. From the depth recovery perspective, (a) shows the projection distribution of 4D radar point clouds on the image plane, which serve as metric anchors for subsequent depth calibration; (b) presents the relative depth map generated by Depth Anything V2, which contains good structural information but lacks absolute scale; (c) displays the metric depth map after disparity-domain RANSAC fitting and Kalman filter temporal smoothing, successfully recovering the true metric scale. From the detection-layer fusion perspective, (d–f) show comparison results in BEV (Bird’s-Eye View): (d) presents camera detection objects based on YOLOv8 and metric depth map (green boxes), featuring high recall but with depth uncertainty; (e) shows radar DBSCAN clustering detection objects (blue boxes), with high localization precision but lacking target semantic information; (f) displays the detection-layer fusion result (purple boxes), fully demonstrating the complementarity of multi-sensor detection, maintaining both high visual recall and improved localization precision through radar.

#### 4.2.2. Ablation Study

To verify the contributions of the key modules, ablation experiments are conducted on the DBSCAN clustering optimization strategy (recursive cluster splitting + historical static-box guidance), the robust fitting mechanism (RANSAC + adaptive MAD threshold), and the depth temporal smoothing mechanism (Kalman filtering). Table 2 reports results for four configurations: Baseline DBSCAN, in which recursive cluster splitting and historical static-box guidance are removed and only standard DBSCAN is used; *w*/*o* RANSAC, in which RANSAC and the adaptive MAD threshold are removed and least-squares fitting is applied directly in the disparity domain; *w*/*o* smoothing, in which Kalman-filter-based temporal smoothing of the depth parameters is removed; and the complete proposed method. In each ablation setting, only the specified module is removed while the remaining components are kept unchanged.

(1) DBSCAN Clustering Optimization

Comparing baseline DBSCAN and the proposed method, the clustering optimization strategy improves overall MOTA from 69.16% to 77.93%, an increase of 8.77 percentage points. The improvement in FPR is most prominent, with overall FPR decreasing substantially from 14.91% to 8.55%, particularly significant in Scene 3 (from 18.37% to 4.39%). This is because baseline DBSCAN tends to over-aggregate multiple adjacent targets into a single large cluster in dense static scenarios, resulting in abnormally oversized detection boxes that generate numerous false positives. The recursive cluster splitting algorithm progressively decomposes oversized clusters into reasonably-sized sub-clusters through geometric constraints, fundamentally suppressing such false detections. Notably, in Scene 1, baseline DBSCAN achieves a lower FNR (17.86%) than the proposed method (21.71%), because over-aggregated large clusters cover wider areas thereby reducing missed detections, but at the cost of significantly elevated FPR (19.84%), with overall MOTA still lower than the proposed method. Scene 3 (off-road) shows the most significant improvement with MOTA increasing by 18.87 percentage points, benefiting from the historical static box guidance strategy ensuring clustering consistency of roadside barriers and vegetation through temporal constraints, combined with recursive splitting correction of over-clustering, jointly achieving substantial tracking performance improvement.

(2) Robust RANSAC Fitting

Comparing the configuration without RANSAC and the proposed method, introducing RANSAC with adaptive MAD thresholds improves overall MOTA from 61.77% to 77.93%, an increase of 16.16 percentage points, which is the largest performance gap among the ablation experiments. In the *w*/*o* RANSAC configuration, all radar-depth correspondence pairs are fitted directly in the disparity domain using least squares without outlier rejection. Because radar point clouds inevitably contain false alarms, multipath reflections, and other noise sources, these outliers bias the estimated scale factor *s* and offset *t*, resulting in severe distortion in metric depth recovery. Among the metrics, the deterioration in FPR is the most pronounced: overall FPR rises sharply from 8.55% to 20.88%, with Scene 1 increasing from 14.90% to 28.09% and Scene 3 from 4.39% to 24.49%. This is because distorted depth estimates lead to substantial errors in the back-projected 3D target positions, thereby introducing many false detections at both the detection and tracking layers. In addition, IDSWR increases from 0.59% to 0.92%, indicating that depth-induced position jumps exceed the prediction range of the tracking filter and trigger more ID switches. The degradation is particularly severe in the off-road scenario (Scene 3), where MOTA drops from 85.61% to 58.76%, because multipath reflections and ground clutter are more frequent and the proportion of outliers is higher. These results verify the effectiveness of the proposed disparity-domain RANSAC depth calibration method: RANSAC isolates outliers through random sampling and consensus, while the adaptive MAD threshold dynamically adjusts the inlier criterion according to the current noise level.

(3) Depth Temporal Smoothing

Comparing the configuration without temporal smoothing and the proposed method, introducing Kalman filtering improves overall MOTA from 73.00% to 77.93%, while reducing overall FPR from 13.42% to 8.55%. This indicates that although single-frame RANSAC fitting can recover depth, the fitted parameters may still fluctuate from frame to frame under noise, causing target position drift and hence false detections. Temporal smoothing effectively suppresses this high-frequency noise. The effect is particularly evident in Scene 1, where FPR decreases from 22.58% to 14.90%. In the off-road scenario (Scene 3), where pose perturbations caused by rough terrain increase alignment noise, Kalman filtering reduces FPR from 11.77% to 4.39% and improves MOTA from 77.20% to 85.61%, demonstrating that temporal modeling is essential for handling interference in unstructured road environments.

**Figure 8:**
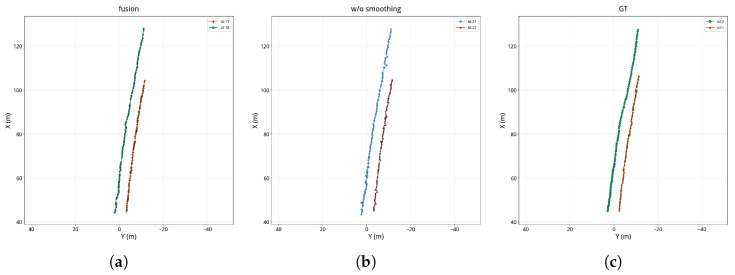
BEV trajectory comparison in Scene 1. (**a**) Proposed method; (**b**) without temporal smoothing; (**c**) ground truth.

**Figure 9:**
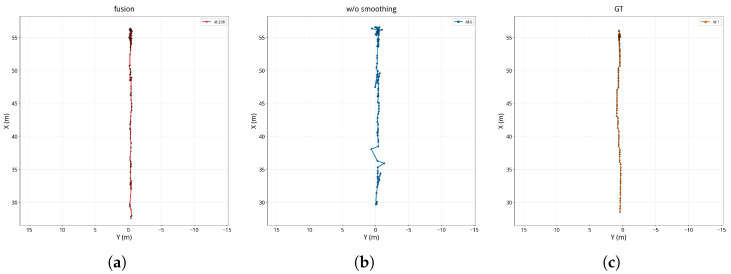
BEV trajectory comparison in Scene 2. (**a**) Proposed method; (**b**) without temporal smoothing; (**c**) ground truth.

**Figure 10:**
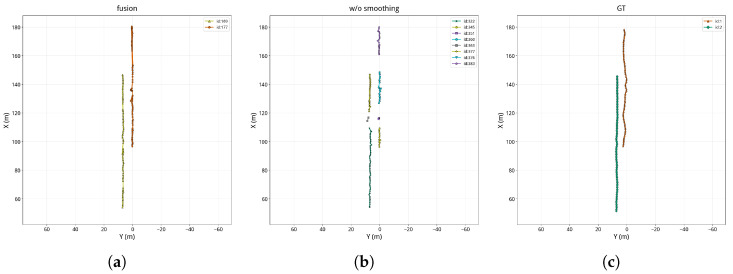
BEV trajectory comparison in Scene 3. (**a**) Proposed method; (**b**) without temporal smoothing; (**c**) ground truth.

To further reveal how temporal smoothing affects tracking stability, Figure 8, Figure 9 and Figure 10 compare the BEV trajectories produced by the proposed method, the method without temporal smoothing, and the ground truth for the three scenes.

The trajectory comparison clearly shows the effect of temporal smoothing on tracking quality. Without temporal smoothing, the per-frame RANSAC-estimated calibration parameters *s* and *t* fluctuate noticeably across frames, leading to poor frame-to-frame consistency in the recovered metric depth maps and causing pronounced zigzag jitter in the back-projected 3D target positions. This positional instability propagates into the tracking layer: when the target position jumps beyond the prediction range of the Kalman filter, matching fails, which in turn causes trajectory fragmentation and ID switches. By contrast, the proposed method temporally smooths the calibration parameters with a Kalman filter, effectively suppressing parameter jitter, yielding smoother and more continuous trajectories that more closely resemble the ground-truth tracks. These observations indicate that temporal smoothing not only reduces FPR but also improves temporal continuity and ID consistency.

## 5. Conclusions

This paper proposes a multi-object tracking framework based on the fusion of 4D millimeter-wave radar and a monocular camera. By exploiting the complementary advantages of the camera’s high recall and the radar’s high localization accuracy, the framework compensates for the respective limitations of sparse 4D radar point clouds and the fact that the radar branch can detect only dynamic targets, as well as the lack of metric depth in monocular vision. In this way, it achieves a complete fusion tracking pipeline without requiring complex 3D annotated data.

The main contributions are threefold. First, a Gaussian distribution-based preprocessing method is adopted to suppress elevation-angle noise in 4D radar point clouds, and, together with velocity compensation and DBSCAN clustering, it enables high-quality radar object detection. In addition, a geometry-constrained recursive cluster splitting algorithm is introduced to address over-clustering, and a historical static-box-guided temporal consistency strategy is proposed to maintain stable detections of static targets across frames. Second, a robust disparity-domain depth calibration method based on RANSAC is proposed. Combined with adaptive MAD thresholds and Kalman-filter-based temporal smoothing, it enables metric recovery of monocular depth. Third, a hierarchical fusion strategy is constructed at the detection and tracking layers, where a linear Kalman filter and the Hungarian algorithm are used to achieve stable multi-object tracking.

Experimental results show that the proposed fusion algorithm achieves an overall MOTA of 77.93% and an overall MOTP of 1.40 m on the self-collected dataset. Compared with the camera-only method, the radar-only method, the conventional IPM-based fusion method, and the point-cloud-projection-based fusion method, the proposed framework improves MOTA by 11.34, 14.79, 31.47, and 30.33 percentage points, respectively, thereby validating the effectiveness of the fusion framework. The ablation study further shows that DBSCAN clustering optimization substantially reduces false positives by suppressing over-aggregation, robust RANSAC fitting guarantees accurate depth recovery by rejecting outliers, and Kalman-filter-based temporal smoothing improves tracking stability by mitigating frame-to-frame parameter fluctuations. Together, these three components enable robust performance in both urban and off-road scenarios.

Future work will focus on three directions: (1) adopting more advanced radar-vision fusion depth estimation networks to further improve the accuracy and robustness of metric depth; (2) introducing more advanced motion models to better handle non-uniform target motion; and (3) exploring end-to-end deep learning-based 3D object detection to further improve overall performance.

## Figures and Tables

**Table 1:** Performance comparison of different methods across scenarios. Bold values indicate the best performance for each metric in each scene.

Scene	Method	FNR (%)	FPR (%)	IDSWR (%)	MOTA (%)	MOTP (m)
Scene 1 (Urban)	Camera-only	28.88	13.36	0.79	59.60	1.60
	Radar-only	35.41	**9.79**	0.77	57.08	**1.49**
	IPM-fused	20.92	28.49	0.78	46.95	1.60
	Point-cloud projection	**13.28**	36.00	2.04	36.18	1.50
	Ours	21.71	14.90	**0.55**	**64.15**	1.61
Scene 2 (Urban)	Camera-only	15.20	7.83	0.54	77.15	1.21
	Radar-only	34.90	**3.66**	0.74	62.14	**1.08**
	IPM-fused	29.64	28.76	**0.52**	41.59	1.12
	Point-cloud projection	**3.76**	30.79	1.29	52.18	1.16
	Ours	**6.55**	4.17	0.63	**88.80**	1.18
Scene 3 (Off-road)	Camera-only	23.97	17.94	**0.19**	59.26	1.51
	Radar-only	14.79	**0.48**	0.58	84.31	1.35
	IPM-fused	17.50	25.49	0.47	53.88	**1.14**
	Point-cloud projection	**9.17**	24.43	1.04	60.52	1.16
	Ours	**9.73**	4.39	0.57	**85.61**	1.47
Overall	Camera-only	22.45	11.95	**0.57**	66.59	1.41
	Radar-only	32.31	**5.67**	0.72	63.14	**1.29**
	IPM-fused	23.43	27.91	0.62	46.46	1.33
	Point-cloud projection	**8.78**	31.65	1.39	47.60	**1.29**
	Ours	**13.47**	8.55	0.59	**77.93**	1.40

**Table 2:** Ablation study results. Bold values indicate the best result in each group comparison.

Scene	Method	FNR (%)	FPR (%)	IDSWR (%)	MOTA (%)	MOTP (m)
Scene 1 (Urban)	Baseline DBSCAN	17.86	19.84	**0.51**	61.39	**1.41**
	*w*/*o* RANSAC	19.52	28.09	1.53	47.81	1.47
	*w*/*o* smoothing	**17.23**	22.58	0.85	57.93	1.54
	Ours	21.71	**14.90**	0.55	**64.15**	1.61
Scene 2 (Urban)	Baseline DBSCAN	14.65	6.65	**0.48**	78.87	**1.09**
	*w*/*o* RANSAC	11.53	9.65	0.82	78.29	1.12
	*w*/*o* smoothing	9.93	**3.71**	0.61	86.05	1.10
	Ours	**6.55**	4.17	0.63	**88.80**	1.18
Scene 3 (Off-road)	Baseline DBSCAN	12.89	18.37	0.89	66.74	1.47
	*w*/*o* RANSAC	12.04	24.49	0.75	58.76	1.54
	*w*/*o* smoothing	10.36	11.77	**0.54**	77.20	**1.40**
	Ours	**9.73**	**4.39**	0.57	**85.61**	1.47
Overall	Baseline DBSCAN	15.56	14.91	**0.58**	69.16	**1.31**
	*w*/*o* RANSAC	14.85	20.88	0.92	61.77	1.35
	*w*/*o* smoothing	**12.91**	13.42	0.68	73.00	1.33
	Ours	13.47	**8.55**	0.59	**77.93**	1.40

## Data Availability

The data presented in this study are available on request from the corresponding author. Part of the data are not publicly available due to our laboratory’s confidentiality agreement and policies.

## References

[1] Zang S., Ding M., Smith D., Tyler P., Rakotoarivelo T., Kaafar M.A. (2019). The impact of adverse weather conditions on autonomous vehicles: How rain, snow, fog, and hail affect the performance of a self-driving car. IEEE Veh. Technol. Mag..

[2] Eigen D., Puhrsch C., Fergus R. Depth map prediction from a single image using a multi-scale deep network. Proceedings of the Advances in Neural Information Processing Systems.

[3] Sun S., Petropulu A.P., Poor H.V. (2020). MIMO radar for advanced driver-assistance systems and autonomous driving: Advantages and challenges. IEEE Signal Process. Mag..

[4] Palffy A., Pool E., Baratam S., Kooij J.F., Gavrila D.M. (2022). Multi-class road user detection with 3+ 1d radar in the view-of-delft dataset. IEEE Robot. Autom. Lett..

[5] Wang L., Zhang X., Li J., Xv B., Fu R., Chen H., Yang L., Jin D., Zhao L. (2022). Multi-modal and multi-scale fusion 3D object detection of 4D radar and LiDAR for autonomous driving. IEEE Trans. Veh. Technol..

[6] Ranftl R., Lasinger K., Hafner D., Schindler K., Koltun V. (2020). Towards robust monocular depth estimation: Mixing datasets for zero-shot cross-dataset transfer. IEEE Trans. Pattern Anal. Mach. Intell..

[7] Ranftl R., Bochkovskiy A., Koltun V. Vision transformers for dense prediction. Proceedings of the IEEE/CVF International Conference on Computer Vision.

[8] Yang L., Kang B., Huang Z., Xu X., Feng J., Zhao H. Depth anything: Unleashing the power of large-scale unlabeled data. Proceedings of the IEEE/CVF Conference on Computer Vision and Pattern Recognition.

[9] Yang L., Kang B., Huang Z., Zhao Z., Xu X., Feng J., Zhao H. (2024). Depth anything v2. arXiv.

[10] Ma F., Karaman S. (2018). Sparse-to-dense: Depth prediction from sparse depth samples and a single image. Proceedings of the 2018 IEEE International Conference on Robotics and Automation (ICRA).

[11] Park J., Joo K., Hu Z., Liu C.K., So Kweon I. (2020). Non-local spatial propagation network for depth completion. Proceedings of the European Conference on Computer Vision.

[12] Long Y., Morris D., Liu X., Castro M., Chakravarty P., Narayanan P. Radar-camera pixel depth association for depth completion. Proceedings of the IEEE/CVF Conference on Computer Vision and Pattern Recognition.

[13] Lin J.T., Dai D., Van Gool L. (2020). Depth estimation from monocular images and sparse radar data. Proceedings of the 2020 IEEE/RSJ International Conference on Intelligent Robots and Systems (IROS).

[14] Nabati R., Qi H. Centerfusion: Center-based radar and camera fusion for 3d object detection. Proceedings of the IEEE/CVF Winter Conference on Applications of Computer Vision.

[15] Zheng L., Li S., Tan B., Yang L., Chen S., Huang L., Bai J., Zhu X., Ma Z. (2023). Rcfusion: Fusing 4-D radar and camera with bird’s-eye view features for 3-D object detection. IEEE Trans. Instrum. Meas..

[16] Bai X., Yu Z., Zheng L., Zhang X., Zhou Z., Zhang X., Wang F., Bai J., Shen H.L. (2025). Sgdet3D: Semantics and geometry fusion for 3D object detection using 4d radar and camera. IEEE Robot. Autom. Lett..

[17] Pan Z., Ding F., Zhong H., Lu C.X. (2024). Ratrack: Moving object detection and tracking with 4D radar point cloud. Proceedings of the 2024 IEEE International Conference on Robotics and Automation (ICRA).

[18] Tang X., Cheng X., Xu N. (2024). A robust multi-object tracking method based on 4D millimeter wave radar and monocular vision fusion. IEEE Sens. J..

[19] Kuan S.Y., Cheng J.H., Huang H.W., Chai W., Yang C.Y., Latapie H., Liu G., Wu B.F., Hwang J.N. (2024). Boosting online 3D multi-object tracking through camera-radar cross check. Proceedings of the 2024 IEEE Intelligent Vehicles Symposium (IV).

[20] Tan B., Ma Z., Zhu X., Li S., Zheng L., Huang L., Bai J. (2023). Tracking of multiple static and dynamic targets for 4d automotive millimeter-wave radar point cloud in urban environments. Remote Sens..

[21] Liu J., Ding G., Xia Y., Sun J., Huang T., Xie L., Zhu B. (2024). Which framework is suitable for online 3D multi-object tracking for autonomous driving with automotive 4d imaging radar?. Proceedings of the 2024 IEEE Intelligent Vehicles Symposium (IV).

[22] Cheng L., Sengupta A., Cao S. (2024). Deep learning-based robust multi-object tracking via fusion of mmWave radar and camera sensors. IEEE Trans. Intell. Transp. Syst..

[23] Tan B., Ma Z., Zhu X., Li S., Zheng L., Chen S., Huang L., Bai J. (2022). 3-D object detection for multiframe 4-D automotive millimeter-wave radar point cloud. IEEE Sens. J..

[24] Shi W., Tong P., Bi X. (2025). Moving-Least-Squares-Enhanced 3D Object Detection for 4D Millimeter-Wave Radar. Remote Sens..

[25] Cheng L., Sengupta A., Cao S. (2023). 3D radar and camera co-calibration: A flexible and accurate method for target-based extrinsic calibration. Proceedings of the 2023 IEEE Radar Conference (RadarConf23).

[26] Cheng L., Cao S. (2023). Online targetless radar-camera extrinsic calibration based on the common features of radar and camera. Proceedings of the NAECON 2023-IEEE National Aerospace and Electronics Conference.

[27] Cheng L., Cao S. (2025). Radar-Camera Fused Multi-Object Tracking: Online Calibration and Common Feature. IEEE Trans. Intell. Transp. Syst..

[28] Kramer A., Stahoviak C., Santamaria-Navarro A., Agha-Mohammadi A.A., Heckman C. (2020). Radar-inertial ego-velocity estimation for visually degraded environments. Proceedings of the 2020 IEEE International Conference on Robotics and Automation (ICRA).

[29] Ester M., Kriegel H.P., Sander J., Xu X. A density-based algorithm for discovering clusters in large spatial databases with noise. Proceedings of the Second International Conference on Knowledge Discovery and Data Mining (KDD-96).

[30] Zhang Y., Sun P., Jiang Y., Yu D., Weng F., Yuan Z., Luo P., Liu W., Wang X. (2022). Bytetrack: Multi-object tracking by associating every detection box. Proceedings of the European Conference on Computer Vision.

[31] Casiez G., Roussel N., Vogel D. 1€ filter: A simple speed-based low-pass filter for noisy input in interactive systems. Proceedings of the SIGCHI Conference on Human Factors in Computing Systems.

[32] Sengupta A., Cheng L., Cao S. (2022). Robust multiobject tracking using mmwave radar-camera sensor fusion. IEEE Sens. Lett..

